# Response Surface Methodology (RSM) Optimization on Extraction and Antioxidant Activity Evaluation of Peptide From Enzymatic Hydrolysates of Housefly (*Musca domestica*) Larvae

**DOI:** 10.1002/fsn3.71058

**Published:** 2025-10-09

**Authors:** Jingnan Miao, Chenglu Yu, Xianhe Cheng, Shumin Liu, Junqiang Qiu

**Affiliations:** ^1^ Key Laboratory of Tropical Translational Medicine of Ministry of Education, International Joint Research Center of Human‐Machine Intelligent Collaborative for Tumor Precision Diagnosis and Treatment of Hainan Province, School of Pharmacy/School of Public Health Hainan Medical University Haikou Hainan China; ^2^ Graduate School, Institute of Traditional Chinese Medicine Heilongjiang University of Chinese Medicine Harbin China

**Keywords:** characterization, *Musca domestica* L., optimization, peptide

## Abstract

Response surface methodology (RSM) was applied to determine the optimal enzymatic hydrolysis conditions for obtaining antioxidant peptides from *Musca domestica* (MDPs). The results indicated that neutral protease was the most effective enzyme for producing MDPs. Under the optimized conditions (time: 3.2 h, temperature: 43.0°C, enzyme dosage: 5300.0 U/g, pH: 6.4), the DPPH radical scavenging rate of MDPs reached 70.9% ± 0.2%. The model demonstrated high reliability, as evidenced by the close agreement between experimental and predicted values, with *R* ^2^_adjusted_ and *R* ^2^_predicted_ both exceeding 0.9036. Subsequently, the particle size, zeta potentials, and structural features of MDPs were characterized using ultraviolet–visible (UV) spectroscopy, Fourier transform infrared spectroscopy (FT‐IR), X‐ray diffraction (XRD), and scanning electron microscopy (SEM). The analyses revealed statistically significant differences in the physicochemical properties among MDPs, MDPs_> 10 kDa_, and MDPs_< 10 kDa_. Furthermore, both MDPs_> 10 kDa_ and MDPs_< 10 kDa_ were rich in amino acids associated with antioxidant activity. Compared to MDPs_> 10 kDa_, the MDPs_< 10 kDa_ exhibited superior antioxidant activities, including DPPH radical scavenging, hydroxyl radical scavenging, and reducing power. This study highlights the potential of *M. domestica* ‐derived peptides as natural antioxidants, supporting their future application in supplements, functional foods, and pharmaceuticals, and promoting the sustainable utilization of this resource.

## Graphical abstract

MDPs were obtained using enzymatic hydrolysi, and the enzymolysis conditions were optimized using response surface methodology (RSM), and the preliminary chemical structures of the MDPs were investigated.

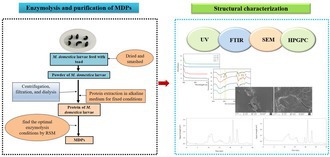

## 1 Introduction

With the acceleration of modern life, humans are increasingly exposed to various harmful stimuli, including poor lifestyle habits, unhealthy diets, and high stress levels (Lee et al. 2024). These factors can lead to the overproduction of free radicals, known as reactive oxygen species (ROS). Excessive accumulation of free radicals induces oxidative stress, which disrupts the body's natural balance and compromises its defense mechanisms (Zhang, Liu, et al. 2024). Oxidative stress damages cells in multiple ways—harming cell membranes, lipids, proteins, and even DNA—thereby accelerating aging and contributing to the development of chronic diseases such as diabetes, cancer, cardiovascular disorders, Alzheimer's disease, and neurodegenerative conditions (Shafras et al. 2024; Hecht et al. 2024; Ren et al. 2024; Guo et al. 2025; Pak et al. 2023). It is noteworthy that DPPH is recognized as an important and prevalent free radical that can adversely affect human cells (Mahmoud et al. 2022). In addition, synthetic antioxidants used in food may pose potential health risks (Yang et al. 2024). Therefore, there is an urgent need to develop safe, potent, and biocompatible natural antioxidants to replace or complement synthetic counterparts. In recent years, efforts to obtain antioxidant peptides from natural sources—such as animal, plant, and insects—have expanded considerably (Shen et al. 2025; Grosso et al. 2025; Yang et al. 2024). There is growing interest in antioxidant peptides due to their high activity, safety, efficient absorption, and hypoallergenic properties. Edible insects represent a promising protein resource for antioxidant peptide production owing to their high protein content (Zhang et al. 2025a; Yang et al. 2025).

There has been a growing interest in finding natural sources of bioactive peptides, which can be obtained through protein hydrolysis and often possess antioxidant activities. In addition, protein hydrolysates usually exhibit higher antioxidant capacity than unhydrolyzed proteins (Tian et al. 2023; De Carvalho Cavenaghi et al. 2025). Studies on animal‐ and plant‐based bioactive peptides have been widely conducted, whereas research on insect‐derived peptides remains relatively limited. Edible insects represent a potential alternative protein source for antioxidant peptide production due to their high protein content. Interest in insect antioxidant peptides has increased in recent years. Unlike synthetic antioxidants (such as butylated hydroxyanisole, butylated hydroxytoluene, and propyl gallate), these natural antioxidant peptides have garnered significant attention owing to their sustainability, low cost, absence of side effects, and natural safety (Abbas et al. 2025; Khasmakhi et al. 2025; Zhang et al. 2025a).

The housefly larvae (*Musca domestica* L., *M. domestica*) are rich in proteins, which account for 58.8% to 63.9% of their dry weight. In traditional Chinese medicine, these larvae are known as “WU GU CHONG” and have been used for centuries to treat childhood malnutrition (Li et al. 2017; Ma et al. 2023; Feng et al. 2010). Surprisingly, despite being the main active components in *M. domestica* , research on *M. domestica* peptides (MDPs) has been limited over the past decade. In general, enzymatic hydrolysis is considered an efficient and safe method for obtaining antioxidant peptides. Compared to chemical methods, enzymatic hydrolysis offers distinct advantages, including mild reaction conditions, precise control, good repeatability, and high selectivity, making it the most commonly used approach for preparing bioactive peptides (Guo et al. 2024; Quan et al. 2025; Wu et al. 2024). Although a limited number of studies have explored the effects of dual‐sweeping‐frequency ultrasound parameters (such as ultrasonic time, power, and frequency) on the antioxidant activities of MDPs during enzymatic hydrolysis, the specific influence of enzymatic hydrolysis conditions on these activities remains unclear (Yang et al. 2024). More significantly, no studies have been reported on the impact of enzyme species on the antioxidant activities of MDPs.

The purpose of this study was to determine the optimal enzymatic hydrolysis parameters (including time, temperature, enzyme dosage, and pH) for obtaining MDPs using response surface methodology (RSM) with a Box–Behnken design (BBD) (Chen et al. 2024; Zhang, Cui, et al. 2024; Leiva‐Portilla et al. 2023). The BBD offers better fitting for quadratic models compared to other experimental designs, along with reduced cost and higher efficiency (Mahmoud and Fawzy 2023). Following this optimization, crude MDPs were purified using ultrafiltration (Cao, Li, Song, et al. 2025; Krunic and Rakin 2025). Multiple advanced analytical techniques were employed to comprehensively characterize the structural properties of the MDPs, including Fourier transform‐infrared spectroscopy (FT‐IR), scanning electron microscopy (SEM), X‐ray diffraction (XRD), and ultraviolet–visible (UV) spectroscopy. Furthermore, a detailed comparative analysis was conducted to evaluate differences among total MDPs, peptides with molecular weight greater than 10 kDa (MDPs_> 10 kDa_), and peptides with molecular weight less than 10 kDa (MDPs_< 10 kDa_). In addition, the in vitro antioxidant activities of the MDPs were assessed using the 2,2‐diphenyl‐1‐picrylhydrazyl (DPPH) radical scavenging assay, hydroxyl radical scavenging assay, and reducing power assay. This groundbreaking study represents the first report on the optimal enzymatic hydrolysis conditions of MDPs from *M. domestica* larvae, making a significant advancement in this field of research.

## 2 Material and Methods

### 2.1 Material

Larvae of *M. domestica* used in this study were purchased in October 2022 from ChanBao Biotechnology Co. Ltd. (Hegang, China). Pepsin, trypsin, neutral protease, papain, alkaline protease, and flavor protease were acquired from Beijing Boao Tuoda Technology Co. Ltd. (Beijing, China). Amino acid standards and 2,2‐diphenyl‐1‐picrylhydrazyl (DPPH) were obtained from Sigma‐Aldrich (St. Louis, MO, USA). Centrifugal filter devices (88513) were supplied by Thermo Fisher Scientific (Waltham, USA). All other reagents were of chromatographic or analytical grade.

### 2.2 Preparation of MDPs

The larvae were processed according to the method of Miao et al. (2024). Briefly, they were cleaned with deionized water and freeze‐dried at −30°C. The dried larvae were then ground into fine particles using a crusher (FW177, Tianjin, China) and sieved through a 40‐mesh sieve. The resulting powder was defatted using petroleum ether to obtain defatted *M. domestica* larvae powder. To extract crude protein, the defatted powder was mixed with alkaline water in a beaker and gently simmered for 2.5 h. The solution was then centrifuged at 3000× g for 15 min at 10°C using a centrifuge (H2050R, Xiangyi Co. Ltd., Changsha, China). The supernatants were collected and stored at −80°C until further use.

For enzymatic hydrolysis, *M. domestica* larvae protein powder (10.0 g) was hydrolyzed under controlled conditions: hydrolysis time (X_1_, 1.0–5.0 h), temperature (X_2_, 25.0°C–65.0°C), enzyme dosage (X_3_, 3000–7000 U/g), and pH (X_4_, 5.0–9.0). After hydrolysis, the enzyme was inactivated by heating at 100°C for 10 min. The solution was neutralized, followed by centrifugation at 4000× g for 10 min. The supernatant was collected, filtered through an ultrafiltration membrane (88,513, Spectrum Medical Industries Inc., Los Angeles, CA, USA) to obtain two fractions: MDPs_> 10 kDa_ and MDPs_< 10 kDa_. The fractions were lyophilized using a freeze dryer (LGJ‐18 N, Yaxingyike Co. Ltd., Beijing, China). The crude MDPs were characterized and stored for further analysis.

### 2.3 Determination of the Appropriate Proteases

Six portions of *M. domestica* larvae protein powder, each weighing 8.0 g, were thoroughly mixed with 120 mL deionized water. The pH of each portion was adjusted to its respective optimal level. Pepsin, trypsin, neutral protease, papain, alkaline protease, and flavor protease were then added individually to each portion at a dosage of 3000 U/g. A seventh portion served as a control and was prepared without the addition of any proteases. Enzymatic hydrolysis was conducted at a constant temperature of 37°C for 4 h with continuous shaking. The reaction was halted by heating the mixtures to 100°C for 10 min (Jiang et al. 2024). The hydrolysates were then filtered through a 0.22 μm membrane filter. The filtrates were collected, freeze‐dried, and stored at −80°C for further use.

### 2.4 Experimental Design of RSM

**FIGURE 1:**
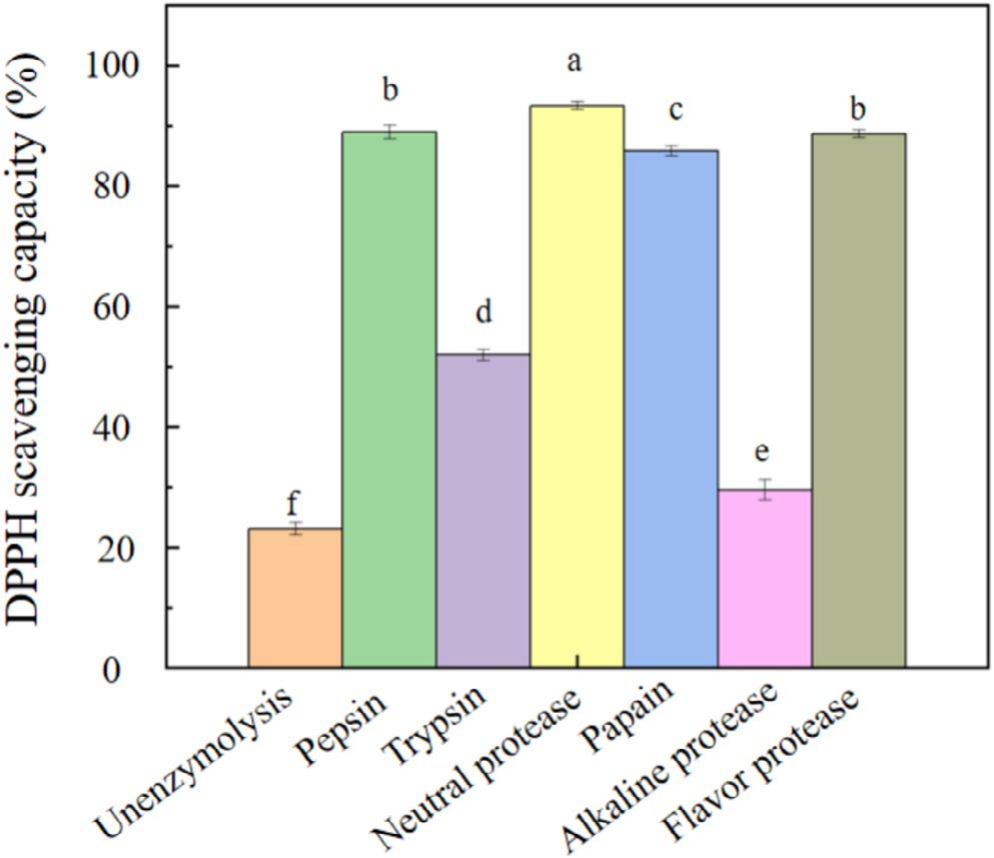
DPPH radical scavenging rate of *M. domestica* protein hydrolyzed with pepsin, trypsin, neutral protease, papain, alkaline protease, and flavor protease. Different lowercase indicates significant difference among protease treatment.

According to the BBD, an extensive RSM investigation was designed. A three‐level (−1, 0, +1) design was employed to determine the optimal enzymatic hydrolysis conditions for MDPs. This involved a detailed study of several parameters using a BBD with four independent variables at three levels, which generated a second‐order polynomial regression model. Based on the analysis of single‐factor test results (Figure 1), the independent variables—hydrolysis time (X_1_), temperature (X_2_), enzyme dosage (X_3_), and pH (X_4_)—were selected. Subsequently, the optimal ranges for these variables were investigated and determined to be suitable for the subsequent tests. The overall design consisted of 29 experimental runs, which intentionally included five replicates at the center points (Table 1). To ensure the validity and robustness of the model, each combination was tested in triplicate.

**TABLE 1:** 4 factor central composite design matrix and the response values for the DPPH radical scavenging (%).

Run	Time (hours) X_1_	Temperature (°C) X_2_	Enzyme dosage (U/g) X_3_	pH X_4_	DPPH radical scavenging activity (%)
1	3.0	45.0	5000.0	6.0	71.6
2	4.0	45.0	5000.0	7.0	68.2
3	2.0	45.0	6000.0	6.0	65.1
4	3.0	45.0	5000.0	6.0	71.6
5	3.0	45.0	4000.0	7.0	68.4
6	4.0	35.0	5000.0	6.0	67.2
7	4.0	55.0	5000.0	6.0	63.1
8	3.0	45.0	6000.0	7.0	68.6
9	3.0	35.0	6000.0	6.0	67.5
10	3.0	55.0	4000.0	6.0	63.9
11	3.0	35.0	4000.0	6.0	65.4
12	3.0	45.0	5000.0	6.0	70.1
13	2.0	45.0	5000.0	5.0	63.6
14	3.0	45.0	4000.0	5.0	67.1
15	3.0	35.0	5000.0	5.0	66.1
16	4.0	45.0	4000.0	6.0	63.1
17	2.0	35.0	5000.0	6.0	63.2
18	2.0	45.0	5000.0	7.0	65.9
19	3.0	45.0	5000.0	6.0	69.9
20	3.0	55.0	6000.0	6.0	64.6
21	2.0	45.0	4000.0	6.0	63.8
22	3.0	55.0	5000.0	7.0	65.6
23	2.0	55.0	5000.0	6.0	60.8
24	3.0	55.0	5000.0	5.0	63.2
25	4.0	45.0	6000.0	6.0	68.3
26	3.0	45.0	6000.0	5.0	67.5
27	3.0	45.0	5000.0	6.0	70.8
28	4.0	45.0	5000.0	5.0	66.9
29	3.0	35.0	5000.0	7.0	68.3

### 2.5 Characterization of MDPs

#### 2.5.1 UV Spectrum and Intrinsic Fluorescence Analysis

First, 10.0 mg of each MDPs sample was dissolved in 5.0 mL of deionized water. The maximum absorption wavelength of each sample (ranging from 200 to 800 nm) was then measured using a U‐2901 UV–vis spectrophotometer (Hitachi Co. Ltd., Tokyo, Japan) (Ji, Hou, et al. 2020; Ji, Yan, et al. 2020). The resulting UV spectra were captured and analyzed using OriginPro 9.0 software.

To further investigate the intrinsic fluorescence properties of MDPs, a solution at pH 7.0 with a concentration of 1.0 mg/mL was prepared in deionized water. The solution was analyzed using an F‐7100 fluorescence spectrophotometer (Hitachi Co. Ltd., Tokyo, Japan). The experimental parameters were set as follows: an excitation wavelength of 280 nm, a scanning speed of 5 nm/s, and an emission spectrum range of 300 to 450 nm at the same scanning speed. A fixed slit width of 5 nm was maintained for both excitation and emission to ensure experimental precision.

#### 2.5.2 Particle Size Distribution and Zeta Potential

The MDPs solutions were diluted to a concentration of 1.0 mg/mL using deionized water, and each diluted sample was transferred into a separate cuvette. Particle size distribution and zeta potential were analyzed using a laser diffraction particle size analyzer (NanoBrook 90Plus, Brookhaven Instruments Corp., New York, USA). All measurements were performed at a constant temperature of 25°C. To ensure accuracy and reproducibility, each sample was tested in triplicate (Han et al. 2025).

#### 2.5.3 FT‐IR Spectra

The infrared spectra of MDPs were recorded using an FT‐IR spectrometer (Nicolet NEXUS 470, Thermo Fisher Scientific, USA). Samples were prepared by homogenizing 10.0 mg of dried MDPs with 100.0 mg of KBr into a fine powder. The mixture was then pressed into pellets with a diameter of 1 mm. Spectra were acquired in transmission mode at room temperature over a wavenumber range of 4000 to 400cm^−1^ (Cao, Li, Chen, et al. 2025).

#### 2.5.4 XRD Analysis

In this study, the XRD patterns of MDPs were acquired following the methodology described by Li et al. (2022) with minor modifications. The crystallinity of MDPs was determined using a wide‐angle X‐ray diffractometer (D8 Advance, Bruker, Germany). The instrument was operated at 40 kV and 40 mA with a Cu Kα radiation source. Data were collected in the 2θ range of 5° to 80° at a scanning speed of 5°/min.

#### 2.5.5 Analysis of Amino Acid Composition

The amino acid composition of MDPs was determined according to the method described by Tian et al. (2022) with slight modifications. Analysis was performed using an automated amino acid analyzer (Model 835–50, Hitachi, Tokyo, Japan). Prior to analysis, the MDPs samples were hydrolyzed with 6 M hydrochloric acid (HCl) at 110°C for 24 h. External standards were used to ensure accurate quantification of the amino acids.

#### 2.5.6 Sem

The freeze‐dried MDPs powder was mounted on specimen stubs using adhesive tape and coated with a gold layer. The micromorphology of the MDPs was observed at magnifications of 200× and 1000× using an EVO18 SEM (Zeiss, Oberkochen, Germany) (Fu et al. 2025).

### 2.6 Measurements of Antioxidant Activity of MDPs

#### 2.6.1 DPPH Scavenging Activity Assay

The DPPH radical‐scavenging ability of MDPs was evaluated according to the method of Gharehbeglou et al. (2024) with minor modifications. Briefly, peptide samples were dissolved in deionized water to obtain solutions at various concentrations. Then, 40.0 μL of each MDPs solution was mixed with 160.0 μL of DPPH ethanol solution (0.4 mM). After thorough mixing, the reaction mixture was incubated for 25 min in the dark. The absorbance was measured at 517 nm using a spectrophotometer (U‐2901, Hitachi Co. Ltd., Tokyo, Japan). Ascorbic acid (Vc) was used as the positive control. The DPPH radical‐scavenging activity was calculated using the following formula:

(1) $$\text{Scavenging activity}\;\left(\%\right)=\left[1-\left(A_{\text{sample}}/A_{\text{blank control}}\right)\right]\times 100$$

where A_blank control_ represents the absorbance of the mixture containing deionized water and DPPH solution, and A_sample_ represents the absorbance of the mixture containing MDPs solution and DPPH solution.

#### 2.6.2 Hydroxyl Radical Scavenging Activity Assay

The hydroxyl radical‐scavenging activity was determined according to the method described by Liu et al. (2023) with some modifications. Briefly, 0.2 mL of MDPs solution at various concentrations was mixed with 0.1 mL of 10 mM FeSO_4_, 0.5 mL of 10 mM salicylic acid‐ethanol solution, and 0.2 mL of 10 mM H_2_O_2_. After thorough mixing, the reaction mixture was incubated at 37°C for 1 h in darkness. The absorbance was then measured at 510 nm using a spectrophotometer (U‐2901, Hitachi Co. Ltd., Tokyo, Japan). Vc was used as the positive control. The hydroxyl radical‐scavenging activity was calculated using the following formula:

(2) $$\text{Scavenging activity}\;\left(\%\right)=\left[1-\left(A_{\text{sample}}/A_{\text{blank control}}\right)\right]\times 100$$

where A_blank control_ represents the absorbance of the blank control group, and A_sample_ represents the absorbance of the sample group.

#### 2.6.3 Reducing Power Assay

The reducing power was determined according to the method of Deng and Huang (2025) with modifications. Briefly, 1.0 mL of MDPs solution at various concentrations was mixed with 1.0 mL of phosphate buffer (pH 6.6) and 1.0 mL of potassium ferricyanide (K_3_Fe(CN)_6_) solution. The mixture was vortexed thoroughly and then incubated in a water bath at 50°C for 25 min. After incubation, 1.0 mL of trichloroacetic acid (TCA, 10% w/v) was added to terminate the reaction. The mixture was then centrifuged, and 2.0 mL of the supernatant was collected. Subsequently, 2.0 mL of deionized water and 0.4 mL of ferric chloride (FeCl_3_, 1% w/v) solution were added to the supernatant. After thorough mixing, the absorbance was measured at 700 nm using a spectrophotometer (U‐2901, Hitachi Co. Ltd., Tokyo, Japan). Vc was used as the positive control.

### 2.7 Statistical Analysis

The data were expressed as mean ± standard deviation (*n* = 3 or *n* = 5, representing the number of samples) and were statistically analyzed using SPSS 13.0 software (IBM, New York, USA). Response surface experiments were conducted using the Design Expert. V8.0.6.1 software. To compare differences between groups, one‐way analysis of variance (ANOVA) followed by Tukey's test was performed using IBM SPSS Statistics 21 software. In addition, either Fisher's *F*‐test or an independent samples *t*‐test was applied where appropriate. A difference was considered statistically significant at *p* < 0.05.

## 3 Results

### 3.1 Determination of the Appropriate Proteases

In the present study, six kinds of proteases—pepsin, trypsin, neutral protease, papain, alkaline protease, and flavor protease—were used to hydrolyze M. domestica larvae protein. The DPPH radical scavenging activity of the hydrolysates was evaluated. The scavenging activities followed the order: neutral protease‐treated group (93.8%) >pepsin‐treated group (89.6%) >flavor protease‐treated group (88.6%) >papain‐treated group (86.8%) >trypsin‐treated group (53.2%) >alkaline protease‐treated group (29.7%) >unhydrolyzed control group (23.2%) (Figure 1).

### 3.2 Influences of Different Enzymatic Hydrolysis Conditions on the DPPH Radical Scavenging Rate of MDPs

**FIGURE 2:**
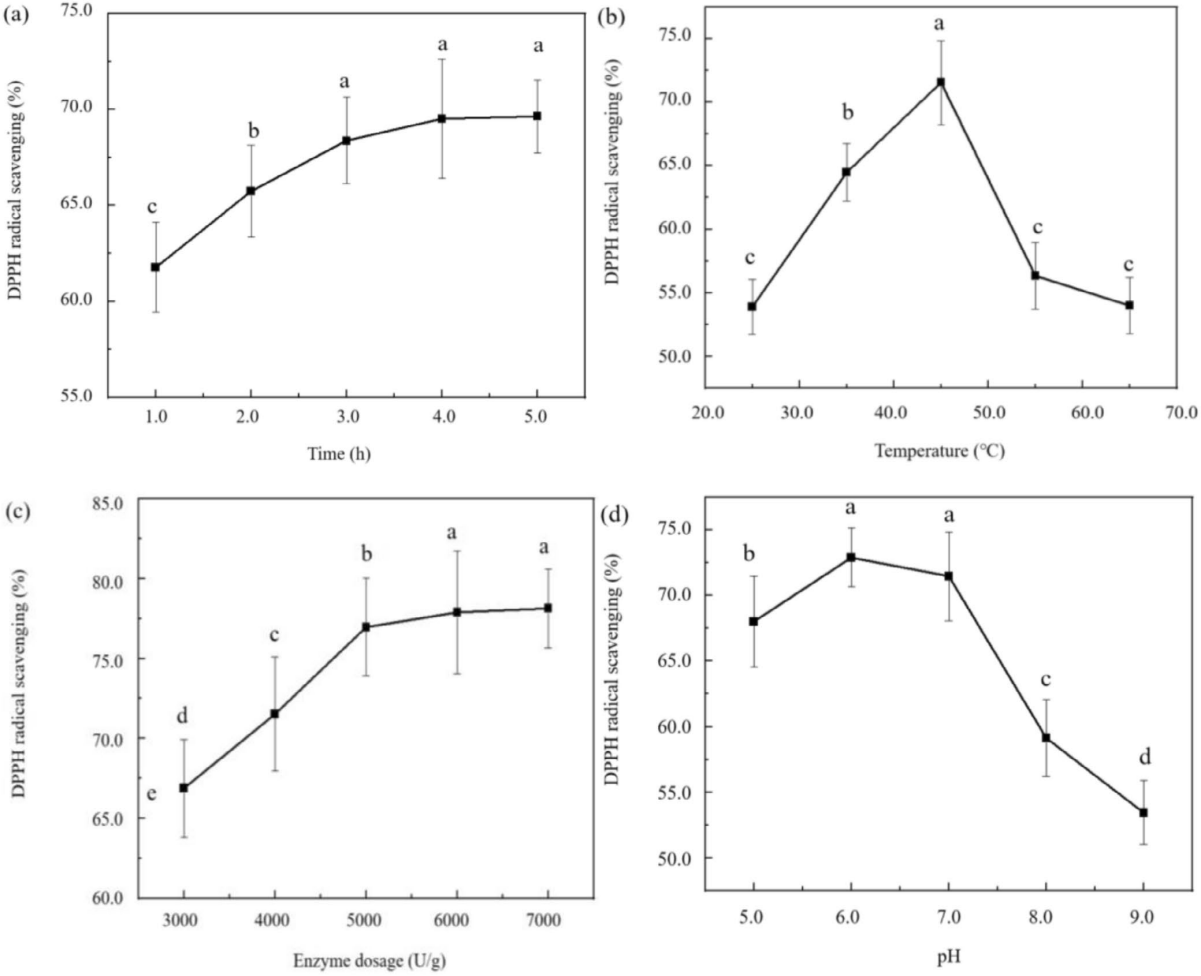
Effects of different extraction parameters on the DPPH radical scavenging rate: (a) Time, h; (b) Temperature, °C; (c) Enzyme dosage, (U/g); (d) pH. Each value represents mean ± SD of triplicates.

The effect of different enzymatic hydrolysis time on the DPPH radical scavenging rate of MDPs (Figure 2a) was first investigated. The experimental conditions were set as follows: temperature maintained at 40.0°C, enzyme dosage at 5000.0 U/g, and pH at 6.0. The results indicated that the DPPH radical scavenging rate of MDPs increased with prolonged hydrolysis time. Specifically, at 4.0 h, a significant increase to 69.5% ± 3.1% was observed. However, beyond this point, a slight decrease in the scavenging rate was detected. Therefore, an optimal hydrolysis time of 4.0 h was selected for subsequent experiments. A detailed assessment of the impact of temperature on the DPPH radical scavenging rate of MDPs was conducted by varying the temperature from 25.0°C to 65.0°C. Other parameters, including time (4.0 h), enzyme dosage (5000.0 U/g), and pH (6.0), were kept constant. The results showed that increasing the temperature from 25.0°C to 45.0°C significantly enhanced the DPPH radical scavenging rate of MDPs (*p* ≤ 0.05), reaching a maximum of 71.6% ± 3.3%. However, further increasing the temperature from 45.0°C to 65.0°C led to a significant decline (*p* ≤ 0.05) in the scavenging rate, as shown in Figure 2b.

To comprehensively evaluate the effect of enzyme dosage on the DPPH radical scavenging rate of MDPs, dosages of 3000.0, 4000.0, 5000.0, 6000.0, and 7000.0 U/g were tested. All other conditions were maintained: hydrolysis time of 4.0 h, temperature of 45.0°C, and pH of 6.0. As illustrated in Figure 2c, the DPPH radical scavenging rate exhibited a consistent upward trend with increasing enzyme dosage, reaching a maximum of 77.0% ± 3.1% at 5000.0 U/g. Beyond this optimal dosage, the scavenging rate increased only marginally.

The influence of pH on the DPPH radical scavenging rate of MDPs was also investigated, as shown in Figure 2d. The pH was adjusted to 5.0, 6.0, 7.0, 8.0, and 9.0, while other parameters—hydrolysis time (4.0 h), temperature (45.0°C), and enzyme dosage (5000.0 U/g)—were strictly controlled. The results revealed a distinct trend: the scavenging rate increased notably between 5.0 and 6.0, then gradually decreased with further increases in pH. The highest DPPH radical scavenging rate of 72.9% ± 2.2% was achieved at pH 6.0.

### 3.3 Optimizing Enzymatic Hydrolysis Parameters of MDPs

#### 3.3.1 Model Fitting

The DPPH radical scavenging rate of MDPs under various experimental conditions and their interactions, along with the corresponding response value, is detailed in Table 1. The effects of different enzymatic hydrolysis parameters—time, temperature, enzyme dosage, and pH—on the DPPH radical scavenging rate of MDPs were evaluated using a BBD. The design consisted of four factors, each at three levels. Five replicates were included at the central point to estimate pure error and assess model stability. Additionally, these center‐point repetitions were used to evaluate the reproducibility and intrinsic variability of the experimental process.

#### 3.3.2 Fitting and Assessing the Adequacy of the Model

Multivariate regression analysis was applied to process the experimental data. A second‐order polynomial model was used to establish the relationship between the independent variable (*X*) and the dependent response variables (*Y*):

(3) $$\begin{array}{cc} Y= & 70.8195+1.1985X_{1}+1.3793X_{2}-0.8264X_{3}+0.8840X_{4}\\ & -0.4288X_{1}X_{2}-0.9730X_{1}X_{3}-0.2586X_{1}X_{4}-0.3545X_{2}X_{3}-0.0496X_{2}X_{4}\\ & -0.0531X_{3}X_{4}-3.6448{X_{1}}^{2}-3.7118{X_{2}}^{2}-1.9018{X_{3}}^{2}-1.1198{X_{4}}^{2} \end{array}$$

where *X*_1_ = Time (hours), *X*_2_ = temperature (°C), *X*_3_ = Enzyme dosage (U/g), and *X*_4_ = pH.

ANOVA results (Table 2) showed that the model had an *F*‐value of 19.76 (*p* ≤ 0.0001), indicating a highly significant correlation with the DPPH radical scavenging rate of MDPs. The lack‐of‐fit test yielded a *p*‐value of 0.4408 (*p* ≥ 0.05), suggesting that the model is well fitted. These results confirm the model's statistical reliability.

**TABLE 2:** Analysis of variance (ANOVA) of the regression parameters.

Parameter	Sum of square	df	Mean square	*F*	*p*	Significance
Model	214.42	14	15.32	19.76	< 0.0001	**
X_1_	17.24	1	17.24	22.24	0.0003	**
X_2_	22.83	1	22.83	29.45	< 0.0001	**
X_3_	8.19	1	8.19	10.57	0.0058	**
X_4_	9.38	1	9.38	12.10	0.0037	**
X_1_X_2_	0.7354	1	0.7354	0.9486	0.3466	—
X_1_X_3_	3.79	1	3.79	4.89	0.0442	*
X_1_X_4_	0.2674	1	0.2674	0.3449	0.5663	—
X_2_X_3_	05027	1	05027	0.6485	0.4341	—
X_2_X_4_	0.0098	1	0.0098	0.0127	0.9120	—
X_3_X_4_	0.0113	1	0.0113	0.0145	0.9057	**
X_1_^2^	86.17	1	86.17	111.16	< 0.0001	**
X_2_^2^	89.37	1	89.37	115.28	< 0.0001	**
X_3_^2^	23.46	1	23.46	30.26	< 0.0001	**
X_4_^2^	8.13	1	8.13	10.49	< 0.0059	**
Residual	10.85	14	0.7752			
Lack of fit	8.25	10	0.8255	1.27	0.4408	Not significant
Pure error	2.60	4	0.6496			CV% = 1.32
*R* ^2^	0.9518		Adjusted *R* ^2^	0.9036		

* Significant at 0.01 < *p* < 0.05.

** Significant at *p* < 0.01.

Furthermore, the adjusted coefficient of determination (*R* ^2^_adj_) was 0.9036, and the coefficient of determination (*R* ^2^) was 0.9518. The coefficient of variation (C.V.) was low (1.32%), demonstrating high reproducibility and reliability of the experimental data. As shown in Table 2, all linear terms (*X*_1_, *X*_2_, *X*_3_, *X*_4_), quadratic terms (*X*_1_^2^, *X*_2_^2^, *X*_3_^2^, *X*_4_^2^), and the interaction terms *X*_1_ *X*_3_ and *X*_3_ *X*_4_ were statistically significant (*p* ≤ 0.05).

#### 3.3.3 Response Surface Analysis

**FIGURE 3:**
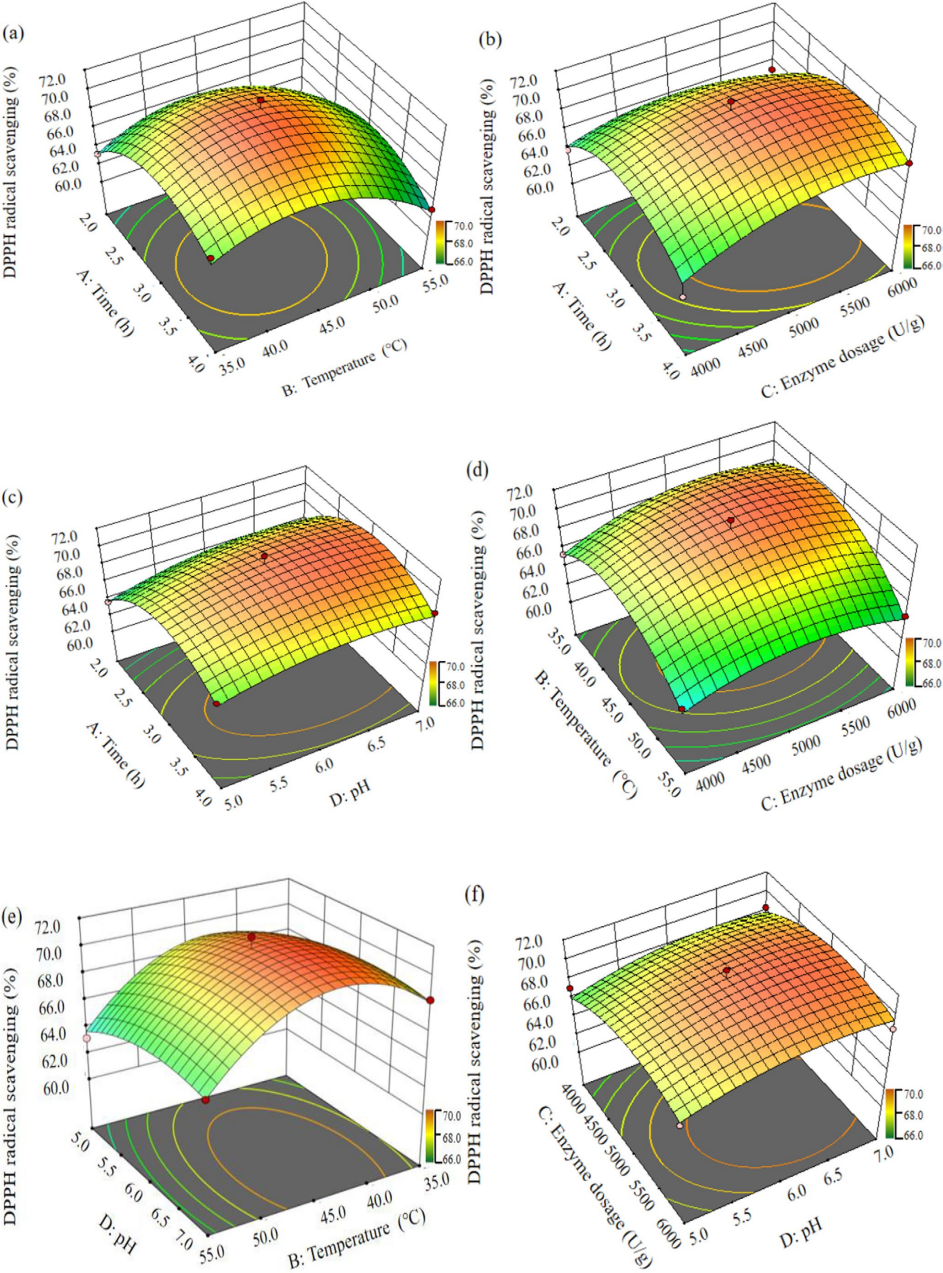
Response surface plots showing the interactions of different selected factors. (a) Effects of time and temperature on DPPH radical scavenging rate; (b) Effects of time and enzyme dosage on DPPH radical scavenging rate; (c) Effects of time and pH on DPPH radical scavenging rate; (d) Effects of temperature and enzyme dosage on DPPH radical scavenging rate; (e) Effects of temperature and pH on DPPH radical scavenging rate; (f) Effects of enzyme dosage and pH on DPPH radical scavenging rate.

**FIGURE 4:**
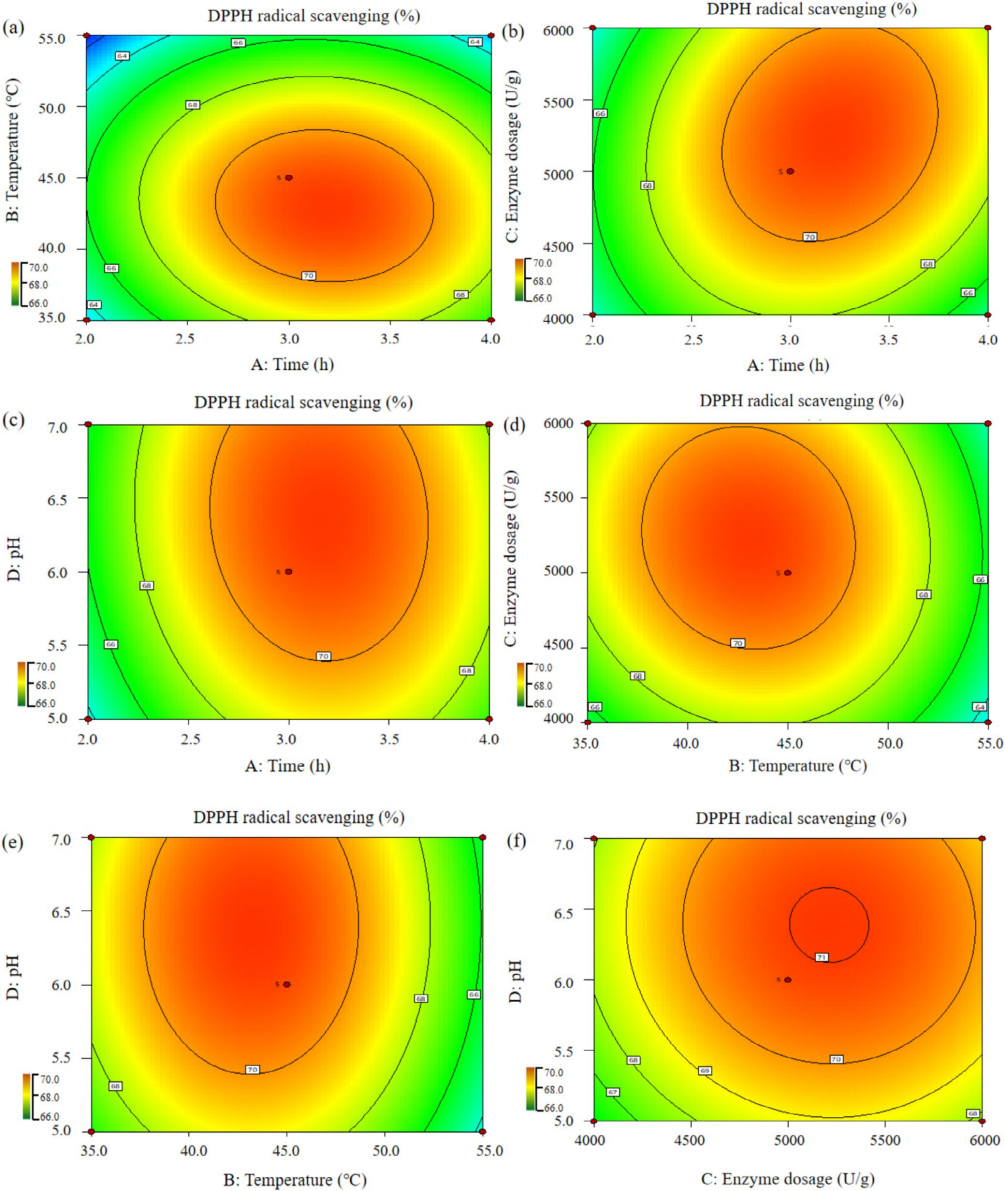
Contour plots displaying the effects of different extraction parameters on DPPH radical scavenging rate. (a) Effects of time and temperature on DPPH radical scavenging rate; (b) Effects of time and enzyme dosage on DPPH radical scavenging rate; (c) Effects of time and pH on DPPH radical scavenging rate; (d) Effects of temperature and enzyme dosage on DPPH radical scavenging rate; (e) Effects of temperature and pH on DPPH radical scavenging rate; (f) Effects of enzyme dosage and pH on DPPH radical scavenging rate.

To gain a comprehensive understanding of the effects of different factors on the outcome, the data were analyzed using the model 3D response surface plots. These plots were carefully examined to identify the optimal values for maximizing the DPPH radical scavenging rate of MDPs. Both 3D response surface plots (Figure 3) and contour plots (Figure 4) were used to accurately elucidate the complex relationships among the variables. The visual elevation observed in the 3D graphs served as an indicator of interactions between each pair of experimental variables.

The combined effects of time and temperature, time and enzyme dosage, and time and pH on the DPPH radical scavenging rate of MDPs are illustrated in Figures 3a–c and 4a–c. These representations revealed that temperature had a more pronounced influence on the scavenging rate compared to time, enzyme dosage, or pH. Specifically, temperature exhibited the most significant impact: an increase from 35.0°C to 45.0°C resulted in a marked exponential rise in the DPPH radical scavenging rate. However, beyond 45.0°C up to 65.0°C, and with pH increasing from 6.0 to 9.0, a substantial decline in the scavenging rate was observed, indicating complex nonlinear interactions among these factors. The effects of temperature and enzyme dosage, as well as temperature and pH, on the DPPH radical scavenging rate are shown in Figures 3d and 4d, and Figures 3e and 4e, respectively. Additionally, when enzyme dosage was increased from 4000.0 to 5000.0 U/g and pH adjusted from 5.0 to 6.0, the scavenging rate gradually improved. The interaction between enzyme dosage and pH was also analyzed and is represented in Figures 3f and 4f.

#### 3.3.4 Model Verification

The optimal conditions for maximizing the DPPH radical scavenging rate of MDPs, as determined by the model, were as follows: time of 3.2 h, temperature of 42.9°C, enzyme dosage of 5283.3 U/g, and pH of 6.4. These conditions were experimentally validated using slightly adjusted practical values: time of 3.2 h, temperature of 43.0°C, enzyme dosage of 5300.0 U/g, and pH of 6.4. Under these optimized conditions, the DPPH radical scavenging rate reached 70.9% ± 0.1%. No significant difference was observed between the predicted value (71.4%) and the experimental results (*p* ≥ 0.05).

### 3.4 Structural Characteristics

#### 3.4.1 Particle Size and Zeta Potential

The particle sizes of MDPs, MDPs_> 10 kDa_, and MDPs_< 10 kDa_ were determined to be 1925.9 ± 49.8, 542.3 ± 62.1, and 5.4 ± 0.6 nm, respectively, as shown in Table 3. Zeta potential, an essential parameter for evaluating the dispersion characteristics of colloidal or polymer solutions, plays a significant role in assessing stability. The absolute zeta potential values of MDPs, MDPs_> 10 kDa_, and MDPs_< 10 kDa_ were −9.9 ± 1.6, −22.5 ± 2.9, and −11.8 ± 1.3, respectively, indicating similar surface charge characteristics across the fractions.

**TABLE 3:** The particle size distribution and zeta potential of MDPs.

Samples	Particle size distribution	Zeta potential
MDPs	1925.9 ± 49.8	−9.9 ± 1.6
MDPs_> 10 kDa_	542.3 ± 62.1**	−22.5 ± 2.9**
MDPs_< 10 kDa_	5.4 ± 0.6**##	−11.8 ± 1.3**##

*Note:* Compared with MDPs, *Significant at 0.01 < *p* < 0.05, **significant at *p* < 0.01. Compared with MDPs_> 10 kDa_, #Significant at 0.01 < *p* < 0.05, ##significant at *p* < 0.01.

#### 3.4.2 Ultraviolet–Visible (UV–vis) Spectra and Intrinsic Fluorescence Spectra

**FIGURE 5:**
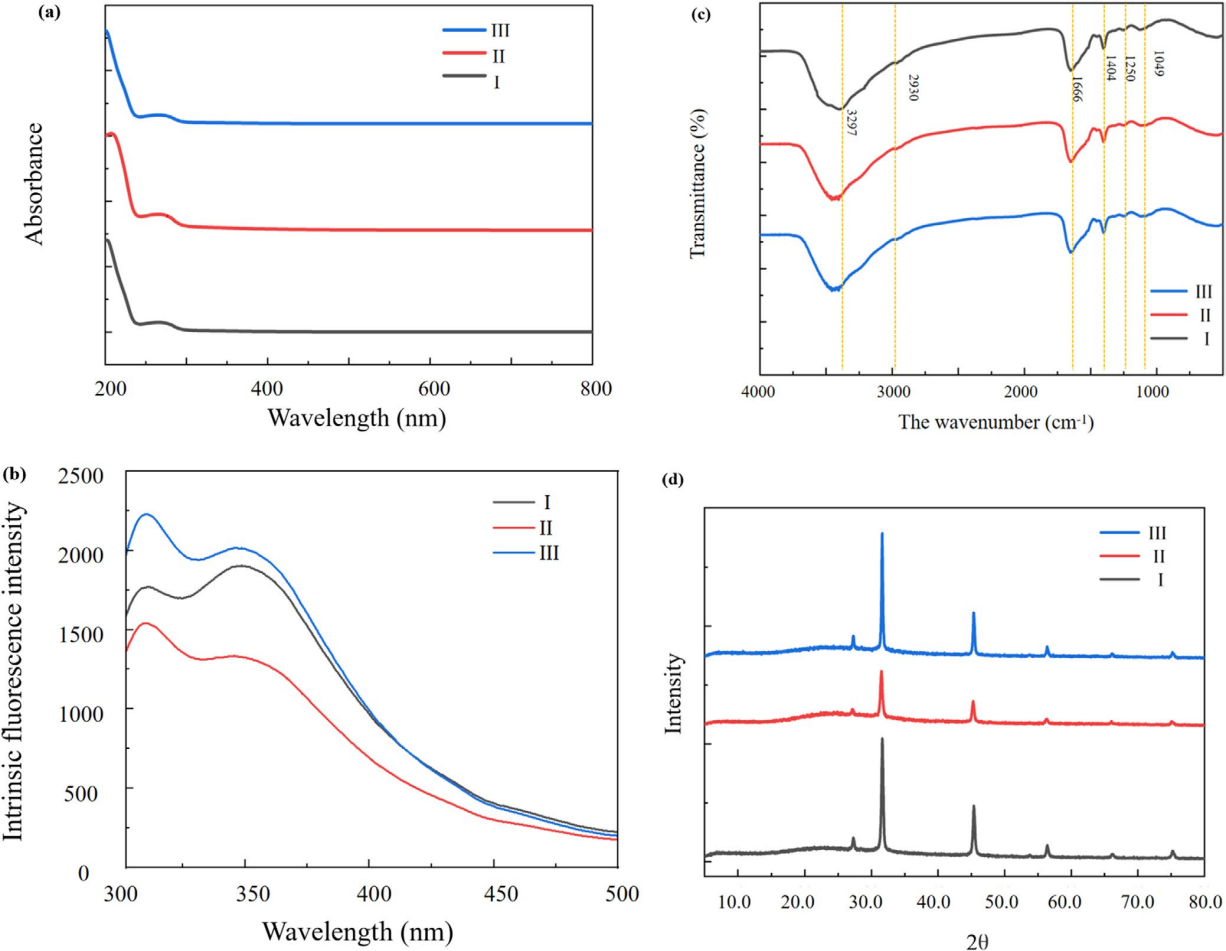
The characterization of MDPs. (a) The UV spectrums of MDPs; (b) Fluorescence spectra of MDPs; (c) The FT‐IR spectra of MDPs; and (d) X‐ray diffraction pattern. I: MDPs, II: MDPs_> 10 kDa_, III: MDPs_< 10 kDa_.

The UV–Vis absorption spectra of MDPs, MDPs_> 10 kDa_, and MDPs_< 10 kDa_ were largely similar, as shown in Figure 5a. The most pronounced absorption for all three samples occurred in the region of 240 to 290 nm. This enhancement resulted in considerably stronger absorption peaks across all fractions. Fluorescence spectroscopy is a suitable technique for detecting subtle structural and conformational changes in the tertiary structures of peptides, particularly when chromophores such as tryptophan, phenylalanine, and tyrosine are present. As shown in Figure 5b, MDPs, MDPs_> 10 kDa_, and MDPs_< 10 kDa_ all exhibited significantly higher fluorescence intensity within the 300 to 375 nm spectral range.

#### 3.4.3 FT‐IR Spectroscopy

FT‐IR analysis is a fundamental method for studying protein and peptide structures. The infrared spectra of the three peptide products were generally similar, as shown in Figure 5c, with detailed data provided in Table 4. FT‐IR distinguishes chemical groups and secondary structures of peptides through characteristic bands, namely amide I (1700 to 1600 cm^−1^), amide II (1550 to 1530 cm^−1^), and amide III (1300 to 1260 cm^−1^) bands. In particular, the amide I band is highly sensitive to secondary structures such as α‐helix, β‐turn, β‐sheet, and random coils. The infrared spectra of all three peptides exhibited considerable similarity. The specific wavenumber positions identified were as follows: (1) Amide A at 3297 cm^−1^ (MDPs) and 3305 cm^−1^ (both MDPs_> 10 kDa_ and MDPs_< 10 kDa_), corresponding to N–H stretching vibration or hydrogen bonding involving N–H groups; (2) Amide B at 2930 cm^−1^; (3) Amide I at 1666 cm^−1^, attributed to C=O stretching vibration or hydrogen bonding associated with carboxyl groups; (4) Amide II at 1404 cm^−1^, indicating N–H bending vibrations and C–N stretching vibrations; and (5) Amide III at 1250 cm^−1^, representing C–N stretching vibrations and N–H deformation vibrations, or vibrations of methylene groups in glycine and proline side chains (Tabarestani et al. 2024). Within the MDPs samples, distinct protein‐related peaks were observed at 3297, 1666, 1404, 1250, and 1049 cm^−1^. The stretching vibrations of N–H and O–H in the range of 3000 to 3750 cm^−1^ were associated with hydrogen bonding within the peptide backbone. These hydrogen bonds play a crucial role in stabilizing peptide secondary structures. Additionally, the absorption peak at 1666 cm^−1^ suggested C=O stretching vibration, while the peak at 1404 cm^−1^ may be assigned to the N–H bending as well as the C–N stretching vibrations.

**TABLE 4:** The secondary structure fitting results of MDPs.

	Content of each component in the Amide I band/%			
	α‐helix	β‐fold	β‐turn	Random coil
MDPs	40.5	32.4	27.1	—
MDPs_> 10 kDa_	42.6	29.8	27.6	—
MDPs_< 10 kDa_	43.1	30.1	26.8	—

Secondary structure analysis derived from FT‐IR data revealed that for MDPs, the predominant conformation was α‐helix (40.5%), followed by β‐fold (32.4%) and β‐turn (27.1%), as summarized in Table 4. For MDPs_> 10 kDa_, the α‐helix content was highest (42.6%), with β‐fold (29.8%) and β‐turn (27.6%) following. Similarly, MDPs_< 10 kDa_ showed a dominant α‐helix structure (43.1%), accompanied by β‐fold (30.1%) and β‐turn (26.8%). Overall, the secondary structures of MDPs, MDPs_> 10 kDa_, and MDPs_< 10 kDa_ were highly similar, indicating that the structural characteristics were well preserved throughout the separation process. This detailed analysis highlights the complex structural organization of MDPs and provides valuable insight into their molecular architecture and functional properties.

#### 3.4.4 Wide‐Angle XRD Experiment

XRD analysis is a widely used method to determine whether particles are crystalline or amorphous, and it is frequently employed to examine the structural conformation of peptides. In this study, XRD was employed to provide a precise determination of the crystal structure of MDPs, MDPs_> 10 kDa_, and MDPs_< 10 kDa_. Distinct diffraction peaks were observed at 2θ values of 26°, 32°, 46°, and 57°, as shown in Figure 5d. The similarity in diffraction patterns suggests that the crystal structures of MDPs, MDPs_> 10 kDa_, and MDPs_< 10 kDa_ are comparable. Notably, all three samples exhibited a prominent diffraction peak, indicating high crystallinity and a well‐organized structure.

#### 3.4.5 Amino Acid Analysis of MDPs

The amino acid composition is a crucial determinant of natural peptide quality. The proportions of 17 amino acids in MDPs were precisely analyzed, as shown in Table 5. The functional characteristics of a protein or peptide are primarily determined by its amino acid content. No significant differences were observed in the amino acid contents among MDPs, MDPs_> 10 kDa_, and MDPs_< 10 kDa_. Both fractions exhibited high nutritional value, making them remarkable sources of protein. MDPs were rich in aspartic acid, glutamic acid, and tryptophan. Additionally, both MDPs_> 10 kDa_ and MDPs_< 10 kDa_ were abundant in glutamic acid, histidine, tryptophan, phenylalanine, and lysine.

**TABLE 5:** The results of amino acid composition analysis of MDPs.

Amino acid	MDPs (mg/mL)	MDPs_> 10 kDa_(mg/mL)	MDPs_< 10 kDa_(mg/mL)
Aspartic acid	0.328	n.d.	0.651
Glutamic acid	0.503	1.095	1.076
Serine	0.126	0.288	0.260
Glycine	0.106	0.236	0.300
Histidine	0.239	0.585	0.675
Arginine	0.145	0.296	0.379
Threonine	0.117	0.296	0.247
Alanine	0.123	0.271	0.477
Tyrptophan	0.324	0.619	0.681
Valine	0.186	0.363	0.383
Methionine	0.110	0.182	0.137
Leucine	0.211	0.396	0.361
Isoleucine	0.139	0.273	0.251
Phenylalanine	0.290	0.534	0.494
Lysine	0.238	0.489	0.478
Essential amino acids	1.292	2.629	2.548
Total amino acids	2.354	4.828	5.123

*Note:* n.d. represents not detected.

#### 3.4.6 SEM Analysis of MDPs

**FIGURE 6:**
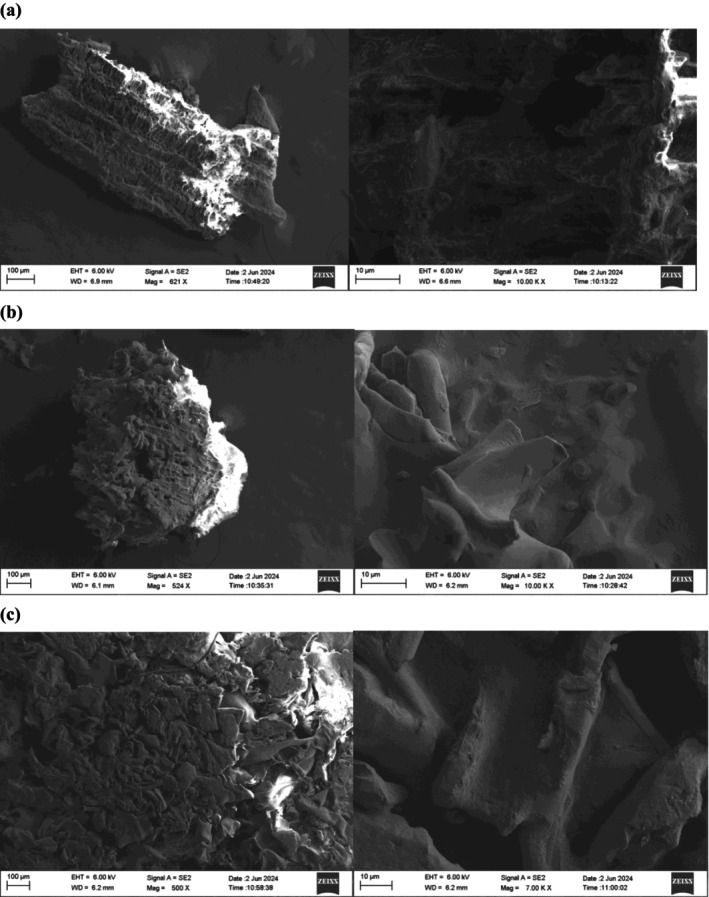
SEM analysis of MDPs (a) MDPs; (b) MDPs_> 10 kDa_; (c) MDPs_< 10 kDa_.

SEM is a commonly employed method for analyzing the microstructure of peptides. The morphology of MDPs, MDPs_> 10 kDa_, and MDPs_< 10 kDa_ was extensively examined using SEM, as shown in Figure 6. Significant differences in the size and surface morphology of these materials were observed. At 1000× magnification, the particles of MDPs, MDPs_> 10 kDa_, and MDPs_< 10 kDa_ exhibited a remarkable resemblance to fragmented glass.

### 3.5 The Antioxidant Activities of MDPs

**FIGURE 7:**
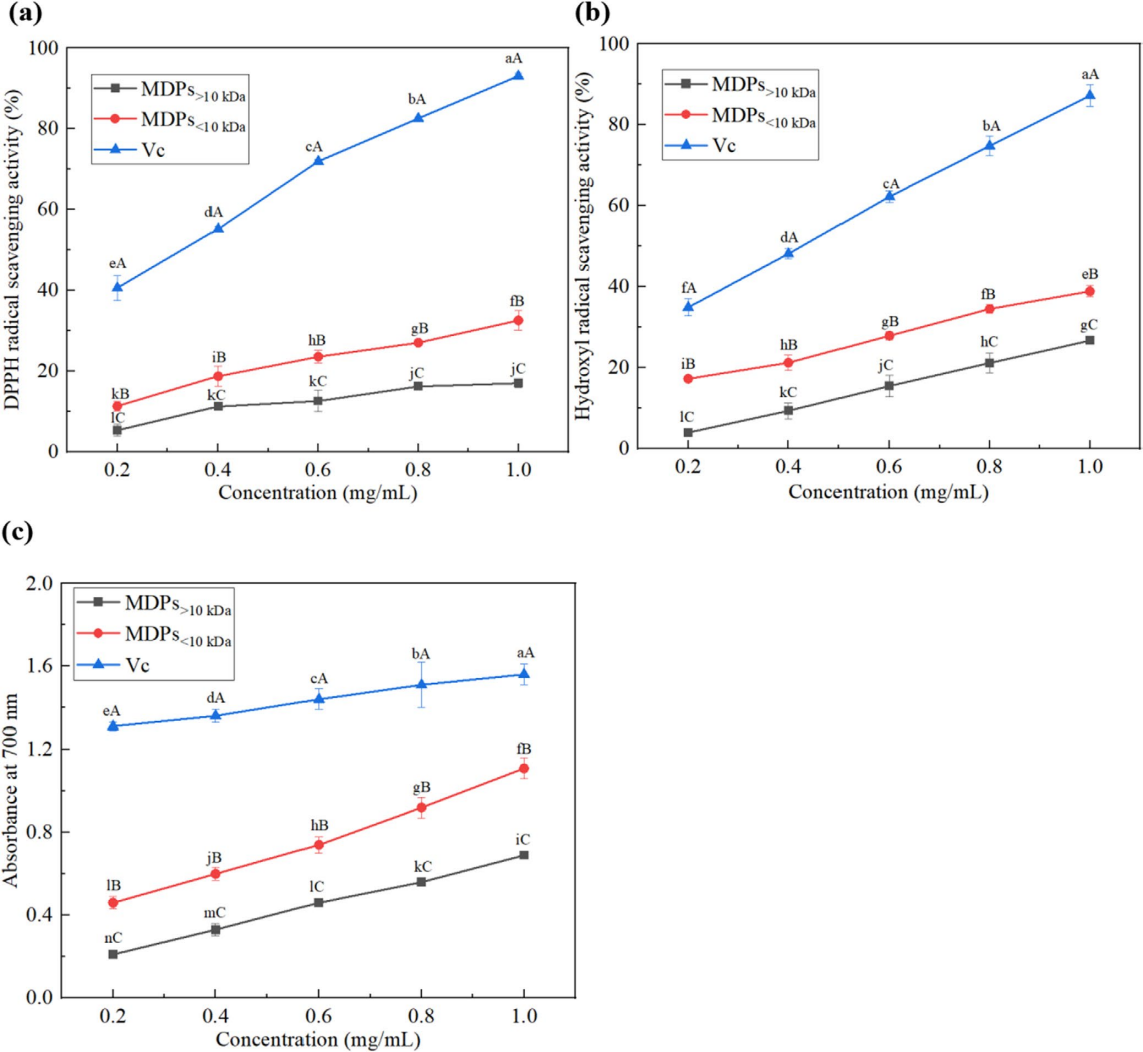
Antioxidant activities of MDPs and Vc at varying concentrations. (a) DPPH radical‐scavenging activity; (b) Hydroxyl radical‐scavenging activity; (c) Reducing power. Means in the same column with different upper‐case letters are significantly different (*p* < 0.05). Means in the same row with different lower‐case letters are significantly different (*p* < 0.05). Bars represent standard deviations from triplicate determinations.

The DPPH radical scavenging activity, hydroxyl radical scavenging activity, and reducing power were used as indices to evaluate the antioxidant activities of MDPs (Li et al. 2022). As shown in Figure 7a, Vc exhibited strong DPPH radical scavenging activity, with rates greater than 40% at all measured concentrations. The DPPH radical scavenging activities of both MDPs_> 10 kDa_ and MDPs_< 10 kDa_ increased in a dose‐dependent manner, ranging from 5.3% to 17.0% and 11.4% to 32.6%, respectively.

As shown in Figure 7b, Vc also showed strong hydroxyl radical scavenging activity, with rates exceeding 35% across the tested concentrations. Similarly, the hydroxyl radical scavenging activities of MDPs_> 10 kDa_ and MDPs_< 10 kDa_ increased in a dose‐dependent manner, within ranges of 4.0% to 26.7% and 17.3% to 38.9%, respectively.

As shown in Figure 7c, Vc demonstrated significant reducing power, with absorbance values at 700 nm exceeding 1.2 across the tested concentrations. The reducing power of MDPs_> 10 kDa_ and MDPs_< 10 kDa_ also increased in a dose‐dependent manner, ranging from 0.21 to 0.69 and from 0.46 to 1.11, respectively.

## 4 Discussion

Enzymatic hydrolysis can disrupt protein structures and release active amino acid residues, thereby enhancing antioxidant activity. Moreover, it can modify the length and composition of small peptides by introducing specific functional groups (e.g., O–H, C–O) (Zhang, Lian, et al. 2024). Compared to the unhydrolyzed group, all six hydrolysates exhibited higher DPPH radical scavenging activity. Enzymatic hydrolysis cleaves peptide bonds, shortens peptide chains, reduces molecular weight distribution, and increases free amino acid content. These changes ultimately improve functional properties, particularly antioxidant activities. The neutral protease hydrolysates showed the highest DPPH radical scavenging rate. Therefore, neutral protease was selected for subsequent analyses. These results are consistent with those reported by Wang et al. (2023) and Meng et al. (2025).

Four critical parameters—enzymatic hydrolysis time, temperature, enzyme dosage, and pH—were investigated using single‐factor experiments, which established a suitable range for BBD. Evidently, enzymatic hydrolysis time had a substantial impact on the DPPH radical scavenging rate of MDPs. The scavenging rate increased when hydrolysis duration was less than 4.0 h and then plateaued thereafter. This stabilization may be attributed to protease inhibition caused by the accumulation of hydrolyzed products and a decrease in available protein substrate, ultimately limiting the generation of antioxidant peptides (Zhang, Fang, et al. 2024). As hydrolysis temperature increased, the DPPH scavenging rate of MDPs also rose. However, beyond 45°C, it declined. This reduction was due to the inactivation of neutral proteases at higher temperatures, which lowered the degree of hydrolysis. Therefore, 45°C was selected as the optimal temperature for subsequent hydrolysis processes (Ding et al. 2025). The DPPH scavenging rate of MDPs increased in a dose‐dependent manner with higher enzyme dosage, consistent with previous reports (Yang et al. 2021). These findings indicate that enzymatic hydrolysis efficiency is correlated with the amount of enzyme used. Similarly, the DPPH scavenging rate increased with rising pH until a value of 6.0, beyond which it decreased. This pattern can be explained by the fact that neutral protease activity reaches its peak within a specific pH range, enabling effective hydrolysis of MDPs and maximizing antioxidant activity. In summary, enzymatic hydrolysis can be modulated by adjusting enzyme type and reaction conditions—such as substrate composition, time, temperature, enzyme concentration, and pH. Nevertheless, the high cost of this method remains a major limitation for its large‐scale application (Luo et al. 2025).

Regarding the DPPH scavenging rate of MDPs, the model demonstrated a strong correlation between theoretical and experimental values. Notably, the lack‐of‐fit test was not statistically significant (*p* ≥ 0.05), indicating that the model effectively captured the underlying relationships within the actual data without significant unexplained variance. This suggests that the model is robust and minimally affected by noise interference. Therefore, the model was considered appropriate for predictive purposes. This comprehensive analysis underscored the complex relationship between the DPPH scavenging activity of MDPs and the enzymatic hydrolysis parameters. Based on *F*‐value analysis, the influence of individual factors on the DPPH scavenging rate decreased in the following order: temperature > time > pH > enzyme dosage. Furthermore, the interaction effects were ranked as follows: *X*_1_ *X*_3_ ≥ *X*_1_ *X*_2_ ≥ *X*_2_ *X*_3_ ≥ *X*_1_ *X*_4_ ≥ *X*_3_ *X*_4_ ≥ *X*_2_ *X*_4_.

Response surface analysis indicated that temperature had a more pronounced effect on the DPPH scavenging rate of MDPs than enzyme dosage and pH, as evidenced by the significantly steeper curvature of its corresponding response surface. The scavenging rate increased rapidly as the temperature rose from 35.0°C to 45.0°C. Furthermore, an increase in the interaction factors led to a progressive enhancement of the scavenging activity. The significance of these interactive effects, reflected in the steepness of the response surfaces, was consistent with the ANOVA results. No significant differences were observed between the predicted and experimental values, strongly validating the model accuracy and its utility in predicting optimal extraction conditions for maximizing MDPs yield.

According to particle size and zeta potential analyses, ultrafiltration reduced the size of MDPs particles by 71.8% and 99.7%, respectively. This decrease in particle size could significantly alter the conformational and functional properties of the peptides. Additionally, the MDPs exhibited a less negative zeta potential compared to other samples. A lower zeta potential reduces electrostatic repulsion between molecules, which may promote their assembly into larger peptide complexes. It also suggests poorer colloidal stability of the MDPs fraction.

Tryptophan, tyrosine, and phenylalanine are aromatic amino acids that confer UV absorption and intrinsic fluorescence properties to proteins and peptides. The UV absorption spectrum of MDPs showed a peak at 270 nm, which is characteristic of these aromatic residues. A similar phenomenon was observed in soy protein by Banerjee et al. (2022). Interestingly, a red shift in the maximum UV absorption wavelength was observed for both MDPs and MDPs_< 10 kDa_, from 337 nm to 340 nm. Additionally, the intrinsic fluorescence of the peptides serves as another indicator of the conformational state of these aromatic residues.

FT‐IR analysis revealed characteristic absorption peaks at 3297, 1666, and 1250 cm^−1^, which are associated with amide A (N–H stretching), amide I (C=O stretching), amide II, and amide III (C–N stretching and N–H bending) vibrational modes, respectively. These bands are inherent to the peptide backbone. These findings are consistent with previous reports (Banerjee et al. 2022; Zhang, Gui, et al. 2024; Samimiazad et al. 2025).

Furthermore, XRD analysis indicated that the MDPs possessed a crystalline structure, a result consistent with the findings of Xu et al. (2023). This agreement provides additional validation for the structural properties of the MDPs.

Based on the amino acid profile, glutamic acid was the most prevalent amino acid in both MDPs and MDPs_< 10 kDa_. However, the proportions of essential amino acids (EAAs) differed significantly between them. The EAA contents of MDPs, MDPs_> 10 kDa_, and MDPs_< 10 kDa_ were 54.89%, 54.45%, and 49.74%, respectively, all exceeding the 40% threshold recommended by the FAO/WHO. Furthermore, the total amino acid content in both MDPs_> 10 kDa_ and MDPs_< 10 kDa_ was higher than that in the MDPs, with the former being nearly twice that of the latter. Literature suggests that positively charged amino acid residues in peptides are essential for antioxidant activity, as they can scavenge radicals by donating protons to reactive oxygen species (ROS). Additionally, hydrophobic amino acids (e.g., valine, isoleucine, leucine, and methionine) are known to enhance the antioxidant activities of peptides. Given their favorable amino acid profile, the MDPs demonstrate high nutritional value and potent antioxidant properties, indicating their potential as a high‐quality dietary protein supplement and source of natural antioxidants.

SEM revealed distinct morphological differences among MDPs, MDPs_> 10 kDa_ and MDPs_< 10 kDa_. At lower magnification, the MDPs exhibited a uniformly dense and rough surface texture. In contrast, both MDPs_> 10 kDa_ and MDPs_< 10 kDa_ appeared as fragmented segments under the same viewing conditions. At a higher magnification (10,000×), the surfaces of MDPs appeared uneven with variable thickness. Notably, all samples contained substantial, irregularly shaped particles with diameters larger than 10 μm. These SEM observations provide detailed insights into the diverse surface morphologies of the MDPs fractions.

The DPPH radical, a stable uncharged molecule, is widely used to assess the radical‐scavenging capacity of antioxidants through its ability to accept hydrogen atoms or electrons (Mahmoud et al. 2022). A reduction in its absorbance upon reaction with antioxidants indicates scavenging activity (Zhang, Liu, et al. 2024). Both MDPs_> 10 kDa_ and MDPs_< 10 kDa_ exhibited significantly weaker DPPH radical scavenging activity compared to Vc. However, MDPs_< 10 kDa_ showed markedly stronger activity than MDPs_> 10 kDa_—more than twice that of the latter. Similarly, both MDPs_> 10 kDa_ and MDPs_< 10 kDa_ had much weaker hydroxyl radical scavenging activity than Vc, but MDPs_< 10 kDa_ demonstrated 1.5 times greater activity than MDPs_> 10 kDa_. At a concentration of 1 mg/mL, the reducing power of MDPs_< 10 kDa_ (1.11) was higher than that of MDPs_> 10 kDa_ (0.69), though both were lower than that of Vc (1.56). These findings validate the excellent antioxidant capabilities of MDPs_< 10 kDa_ against various free radicals and suggest their potential for broader application in mitigating oxidative stress. The results also indicate that peptides with lower molecular weight generally possess stronger antioxidant activities, which was consistent with the results of other research (Zhang, Cui, et al. 2024). Recently, antioxidant hydrolysates and peptides have attracted considerable attention due to their potential to reduce oxidative stress with a better safety profile compared to synthetic antioxidants and medications. As a result, antioxidant peptides derived from natural proteins are increasingly recognized and studied.

The antioxidant activities of peptides are significantly influenced by their amino acid composition, particularly residues such as aspartic acid, glutamic acid, arginine, alanine, glycine, proline, leucine, valine, and tyrosine (Zhang, Lian, et al. 2024; Wang et al. 2023; Li et al. 2022). Additionally, tryptophan, tyrosine, phenylalanine, and proline can quench free radicals by donating protons to electron‐deficient radicals (Leiva‐Portilla et al. 2023). The current study found high proportions of aspartic acid, glycine, arginine, alanine, and valine, as presented in Table 5. Aspartic acid, for example, can scavenge free radicals due to its surplus electrons. Moreover, molecular weight is a critical factor affecting peptide antioxidant activity. In this study, MDPs_< 10 kDa_ exhibited higher DPPH radical scavenging, hydroxyl radical scavenging, and reducing power than MDPs_> 10 kDa_. These results are consistent with the findings of Wang et al. (2025), who reported that dry‐cured beef peptide fractions below 10 kDa had significantly greater antioxidant activities than higher molecular weight fractions. The enhanced antioxidant effects of low‐molecular‐weight peptides may be attributed to their improved ability to chelate transition metals and scavenge free radicals (De Carvalho Cavenaghi et al. 2025).

## 5 Conclusion

In this study, the DPPH radical scavenging rate of MDPs significantly increased from 54.6% to 71.6% under optimal hydrolysis conditions (time: 3.2 h, temperature: 43.0°C, enzyme dosage: 5300.0 U/g, and pH: 6.4). The physical and chemical characteristics of MDPs, MDPs_> 10 kDa_, and MDPs_< 10 kDa_ were found to be highly similar, as confirmed by UV, FT‐IR, XRD, and SEM analyses. Furthermore, comprehensive amino acid analysis revealed high concentrations of both essential and total amino acids in both MDPs_> 10 kDa_ and MDPs_< 10 kDa_. The stronger antioxidant activity observed in MDPs_< 10kDa_ may be attributed to their lower molecular weight and specific amino acid composition. These results underscore the potential of MDPs as a promising source of bioactive peptides. Overall, enzymatic hydrolysis effectively enhanced the antioxidant activity of the peptides, modified their structure, and improved functional properties. This study provides a solid foundation for developing novel antioxidant ingredients from insect proteins for applications in the food and pharmaceutical industries. Further research on the purification and antioxidant mechanisms of MDPs is warranted.

## Author Contributions

**Jingnan Miao:** conceptualization (equal), methodology (equal), writing – original draft (equal). **Chenglu Yu:** formal analysis (equal), investigation (equal). **Xianhe Cheng:** writing – review and editing (equal). **Shumin Liu:** funding acquisition (equal), project administration (equal), supervision (equal).

## Conflicts of Interest

The authors declare no conflicts of interest.

## Data Availability

The data presented in the study is available in the article.
